# Pathologic assessment of resected stage III non‐small cell lung cancer after neoadjuvant chemotherapy: identification of additional prognostic factors

**DOI:** 10.1111/his.70025

**Published:** 2025-11-09

**Authors:** Francesca Lunardi, Alessandra Ferro, Luca Vedovelli, Federica Pezzuto, Sofia‐Eleni Tzorakoleftheraki, Asuman Kilitci, Yuliia Kuzyk, Simone Zanella, Marco Schiavon, Federico Rea, Giulia Pasello, Fiorella Calabrese

**Affiliations:** ^1^ Department of Cardiac, Thoracic, Vascular Sciences and Public Health University of Padova Padova Italy; ^2^ Division of Medical Oncology 2 Veneto Institute of Oncology – IRCCS Padova Italy; ^3^ Department of Pathology Aristotle University of Thessaloniki Thessaloniki Greece; ^4^ Department of Medical Pathology, Faculty of Medicine Düzce University Düzce Turkey; ^5^ Department of Pathology and Forensic Medicine Danylo Halytsky Lviv National Medical University Lviv Ukraine; ^6^ Department of Surgery, Oncology, and Gastroenterology University of Padova Padova Italy

**Keywords:** artificial intelligence, morphometry, neoadjuvant chemotherapy, NSCLC, tumour bed

## Abstract

**Background:**

Non‐small cell lung cancer (NSCLC) patients undergoing neoadjuvant chemotherapy (NACT) followed by surgery represent an ideal clinical setting to identify prognostic factors. To date, major pathological response (MPR) and complete pathological response (pCR) have been used as surrogates of NACT response and clinical outcome. The aim of the study was to investigate the role of additional clinico‐pathological features, taking advantage of morphometry and artificial intelligence (AI).

**Methods:**

Seventy stage III NSCLC patients undergoing surgery after NACT were studied. A granular evaluation of histological parameters with morphometrical quantification of the stromal components (fibrosis/inflammation) in addition to the tumour bed analysis (2020 IASLC statement) was carried out in all cases. An AI algorithm of the different immunophenotypes was also applied on immunohistochemistry‐stained whole‐slide images. A *ClinPATH combined score* including MPR, baseline blood lymphocytes, perineural invasion, vascular invasion, proliferative index, fibrosis extension percentage and AI‐quantified CD4+ cell % was tested.

**Results:**

MPR and pCR were related to disease‐free survival (DFS) and overall survival (OS) but also vascular/perineural/pleural invasion and Ki‐67 were useful in stratifying the study population. Concerning the tumour bed stromal components, only morphometrical quantification highlighted the prognostic role of fibrosis and inflammation, particularly when distinguishing CD4+ and FOXP3+ cells, mainly in adenocarcinomas. Interestingly, the combination of the most impactful clinico‐pathological parameters in a *ClinPATH combined score* correlated better with DFS and OS than any individual parameter, including MPR or pCR.

**Conclusion:**

AI‐based method can be used to accurately decipher the complexity of tumour bed stromal components, providing extra information for outcome prediction. The combination of different clinico‐pathological features could be highly valuable in guiding therapeutic decisions and ultimately improve patient outcomes.

## Graphical abstract

Multiple pathological variables are associated with prognosis in resected NSCLC at stage III undergoing neoadjuvant treatment, namely vascular, perineural and pleural infiltration, proliferation index, pathological response, fibrosis and presence of lymphocytes in the tumour bed. Moreover, when these variables are combined in a clinical‐pathological score, prognostic accuracy improves further.

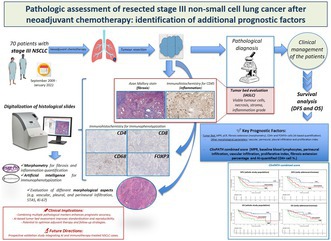

**Abbreviations**
AI: Artificial intelligence
pCR: Complete pathological response
DFS: Disease‐free survival
ICIs: Immune checkpoint inhibitors
irPRC: Immune‐related pathological response criteria
IHC: Immunohistochemistry
IASLC: International Association for the Study of Lung Cancer
MPR: Major pathological response
NACT: Neoadjuvant chemotherapy
NLR: Neutrophil‐to‐lymphocyte
NSCLC: Non-small cell lung cancer
OS: Overall survival
PRSC: Prognostic score for residual cancer
STAS: Spread through air spaces
TME: Tumour microenvironment
UICC: Union for international cancer control
IOV: Veneto Institute of Oncology
WSI: Whole slide image

## Introduction

Approximately 30% of all non‐small cell lung cancer (NSCLC) cases are diagnosed at an early stage that may be considered as radically resectable and therefore potentially curable; among these patients, about 20%–25% present with stage IIIA or IIIB disease.^1^, ^2^, ^3^ Surgery is the primary treatment option for patients with resectable NSCLC and is recommended with neoadjuvant or adjuvant systemic therapy for stages II to IIIB disease.^4^ Neoadjuvant platinum‐based chemotherapy (NACT) followed by resection has been used in patients with locally advanced NSCLC to address the high rate of local and systemic failure.^5^, ^6^ Different studies have shown that histopathologic features in the resected specimens after NACT or chemoradiotherapy, particularly major pathological response (MPR) and complete pathological response (pCR), are associated with significant improvements in disease‐free survival (DFS) and overall survival (OS).^5^ Recently, several randomized clinical trials have investigated the use of neoadjuvant immune checkpoint inhibitors (ICIs) in combination with platinum‐based chemotherapy demonstrating that ICIs can prolong event‐free survival and increase the percentage of pCR.^7^

In 2020, the International Association for the Study of Lung Cancer (IASLC) published recommendations to standardize the pathological evaluation of NSCLC histological specimens after neoadjuvant therapies, supporting the importance of assessing the whole tumour bed, thus not only the percentage of viable tumour cells but also necrosis and stromal components.^8^ In recent years, various studies have been published on the gross processing and microscopic evaluation of lung carcinoma specimens in the neoadjuvant setting, with the ultimate goal of standardizing pathology practices.^8^, ^9^, ^10^, ^11^, ^12^, ^13^, ^14^, ^15^, ^16^ However, certain issues are still debated such as inter‐observer disagreement^9^, ^10^ and the evaluation in different histotypes.^11^

In particular, a challenging task among pathologists is the quantification of stromal components of the tumour bed.^12^ Indeed, the evaluation of inflammation and fibrosis extension is extremely subjective and associated with a low reproducibility, thus affecting its potential prognostic utility in the case of neoadjuvant therapy.^12^

Immune cells within the tumour microenvironment (TME) have been shown to play an important role in the development, progression and outcomes of NSCLC, even in those that are radically resected. In particular, some studies have evaluated total tumour infiltrating lymphocytes, while others have studied specific immune components, alone or in combination.^17^ There is a growing interest in identifying the crucial immune components after the introduction of neoadjuvant PD‐1/PD‐L1 immune checkpoint blocking agents. Cottrell *et al*. developed ‘immune‐related pathological response criteria’ (irPRC) and highlighted for the first time the importance of distinguishing the different inflammatory cell types for prognostic/predictive purposes.^18^

In this study, we aimed to assess the features of histopathological response to NACT, particularly focusing on tumour bed stromal components, with more objective and reproducible methodologies such as morphometry and artificial intelligence (AI). Moreover, we explored the role of additional morphological parameters alone or in combination to accurately predict the prognosis of patients with NSCLC undergoing surgery after NACT.

## Materials and Methods

### Study Population

This is a retrospective, longitudinal, single‐centre study enrolling a consecutive series of stage III NSCLC patients who underwent radical surgery after NACT between September 2009 and January 2022. The inclusion criteria were the following: (1) written informed consent for the study; (2) histologically or cytologically diagnosed with NSCLC; (3) stage III disease radically treated with surgery after platinum‐based chemotherapy.

This study was conducted according to the current rules of Good Clinical Practice and principles of the Helsinki declaration and all the patients gave an informed consent to the study. The Veneto Institute of Oncology (IOV) Ethical Committee approved the study (CESC IOV: 2021–89).

All clinico‐pathological and laboratory data were collected at the time of diagnosis, before neoadjuvant treatment, including histology and disease stage according to the Eighth Edition of the Union for International Cancer Control (UICC) TNM Classification of Malignant Tumours. Primary tumour tissue specimens were classified in accordance with the 2021 WHO classification of Tumours of the Lung, Pleura, Thymus and Heart (fifth edition) and the American Joint Committee on Cancer TNM staging manual (eighth edition). All histopathological features were also recorded, including vascular, perineural and pleural invasion, as well as spread through air spaces (STAS), defined as the presence of tumour cells—either as single cells, micropapillary clusters or solid nests—within the alveolar spaces beyond the edge of the main tumour. The tumour bed was assessed according to the recommendations of IASLC, taking into account the presence of viable tumour cells, necrosis and stroma (that includes inflammation and fibrosis), with the total adding up to 100%.^8^ Inflammation was graded based on tissue involvement as mild (involvement of <30% of the tissue), moderate (involvement of 30–60% of the tissue) and marked (involvement of >60% of the tissue). In the case of adenocarcinomas, we applied the current WHO classification system and defined it as Grade 1 (well‐differentiated) when lepidic‐predominant with no or <20% high‐grade pattern, Grade 2 (moderately differentiated) when acinar or papillary‐predominant with no or <20% high‐grade pattern, and Grade 3 (poorly differentiated) when ≥20% high‐grade pattern (solid, micropapillary, cribriform or complex glandular pattern). PD‐L1 positivity was evaluated as Tumour Proportion Score (TPS) on tumour cells.

Subsequent systemic or locoregional treatments, data of possible relapsing of disease and death were also collected: 32 patients (46%) underwent postoperative radiotherapy, and only three received adjuvant systemic therapies.

### Pathological Response Evaluation

The pathological response was assessed to evaluate the efficacy of NACT. In particular, considering residual viable tumour cells, pathologists evaluated both MPR and pCR. MPR is defined as ≤10% residual viable tumour in the resected lung, while pCR is defined as the complete absence of viable tumour cells in both the resected primary lung tumour and all sampled regional lymph nodes (N0), following neoadjuvant therapy. In cases where the initial microscopic evaluation suggested a pCR, the entire tumour bed was subsequently sampled and thoroughly reviewed.^8^

### Immunohistochemical Study, Morphometry and AI Approach

Azan‐Mallory staining was performed for collagen evaluation and immunohistochemistry (IHC) with monoclonal antibody anti‐CD45 for inflammatory cell infiltrate quantification. The analysis was restricted to the most representative section of the tumour bed, which was defined as the tissue section with a percentage of fibrosis/inflammation similar to the whole tumour bed quantification. In a subset of cases, the analysis was done in all tumour bed sections and there were no significant differences with the ‘hot spot’ quantification (data not shown).

Whole‐slide image (WSI) capture was performed on an Aperio AT2 slide scanner (Aperio Technologies, Leica Biosystems), and computer‐assisted morphometry was used (Image Pro‐Plus).

The regions of interest were initially selected and quantified using dedicated image analysis software; this process was supervised by an experienced pathologist, who verified and confirmed the accuracy and consistency of the selected areas. Both fibrosis and inflammation extension were expressed as a percentage of the total lung tissue area.

Moreover, an inflammatory cell characterization was performed by IHC in the tissue serial sections obtained from the same blocks using monoclonal antibodies anti‐CD4, anti‐CD8, anti‐CD68 and anti‐FOXP3 (all Leica Biosystems, Italy). These markers were chosen because of their role within the TME. Even in this case, WSI capture was performed at 40X magnification, and we authored the application available in Visiopharm's APP Center (https://visiopharm.com/app‐center/) to optimize the detection of the brown‐stained inflammatory cells. The different cell components were expressed as a percentage/total number of cells.

### Statistical Analysis

DFS was calculated from the date of radical surgery to the date of first recurrence of disease and OS from the date of radical surgery until death. Continuous pathological variables were converted into categorical (low/high or low/intermediate/high) using median or quartiles (Q1, median and Q3) as cut‐off values. Association of different variables with response and survival was explored using Cox univariate regression analysis. Survival curves were calculated using the Kaplan–Meier estimator. All analysis was made using the R language and environment version 4.4.1 (R Foundation). Three *ClinPATH combined scores* were tested, and they included MPR, baseline blood lymphocytes, perineural invasion, vascular invasion, proliferative index, fibrosis extension percentage (A), ± AI‐quantified CD4+ cell % (B) and ± AI‐quantified FOXP3+ cell % (C). In adenocarcinomas, the three combined scores were evaluated also after incorporating WHO grading. *ClinPATH composite scores* were categorized according to their empirical distribution, using quartiles and the median/quartiles as reference thresholds. Score A (0–7) was classified as low 0–2, intermediate 3–4, high 5–7; Score B (0–8) as low 0–3, intermediate 4–5, high 6–8; and Score C (0–9) as low 0–3, intermediate 4–5, high 6–9. Given the focus on constructing an interpretable composite index rather than a fully specified predictive model and that the limited number of patients and events was insufficient to support robust multivariable modelling with multiple candidate predictors, we adopted a univariable‐driven selection strategy, combined with biological plausibility, to avoid overfitting and instability of estimates.

## RESULTS

### Pathological Response and Morphological Aspects

Seventy patients were included in the study, and the main clinical characteristics at the time of diagnosis are reported in Table 1.

**Table 1:** Study population characteristics at the time of diagnosis (pretreatment)

Variable*	
Overall population	70 (100)
Gender	
Female	28 (40)
Male	42 (60)
Age at diagnosis	
Years [median (IQR)]	67.5 (61.7–71)
ECOG PS at diagnosis	
0	39 (56)
1	31 (44)
Smoking habit	
Never‐smoker	15 (21)
Former‐smoker	28 (40)
Current‐smoker	27 (37)
Weight loss	
No	59 (84)
Yes	11 (16)
Stage at diagnosis	
IIIA	41 (59)
IIIB	29 (41)
Histology	
Adenocarcinoma	45 (64)
Squamous cell carcinoma	18 (26)
Adenosquamous cell carcinoma	2 (3)
Not otherwise specified	5 (7)
PD‐L1 expression	
<1%	18 (26)
1%–49%	8 (11)
≥50%	7 (10)
Unknown	37 (53)
Neutrophil count at baseline (×10^9^/L) [median (IQR)]	5 (4–7)
Lymphocyte count at baseline (×10^9^/L) [median (IQR)]	2 (2–3)
Platelet count at baseline (×10^9^/L) [median (IQR)]	268 (241–367)
NLR [median (IQR)]	3 (2–4)
PLR [median (IQR)]	139 (114–211)

Abbreviations: ECOG PS, Eastern Cooperative Oncology Group Performance Status; IQR, interquartile range; *N*, number; NLR, neutrophil‐to‐lymphocyte ratio; PLR, platelet‐to‐lymphocyte ratio.

* All data are reported as *N* (%), if not otherwise specified.

In our study population, 12 (17%) patients showed MPR and 5 (7%) showed pCR. The evaluation of the tumour bed showed a median (IQR) necrosis of 5% (1%–19%), while the stromal component extension was 30% (15%–50%). When focusing only on inflammation, the grade was mild in 26 (37%), moderate in 34 (49%) and marked in 10 (14%) patients (Table 2). All other morphological features are reported in Table 2. EGFR status was available in 48/70 patients (68.6%), with mutations detected in 25% of evaluable cases. Testing was not applicable in 25.7% (squamous cell carcinomas) and unavailable in 5.7%.

**Table 2:** Pathological characteristics of NSCLC surgical samples

Variable*	
Overall population	70 (100)
Tumour bed	
Viable tumour % [median (IQR)]	55 (21–70)
Necrosis % [median (IQR)]	5 (1–19)
Stroma % [median (IQR)]	30 (15–50)
Inflammation grade	
Mild	26 (37)
Moderate	34 (49)
Marked	10 (14)
MPR	12 (17)
pCR	5 (7)
PD‐L1 expression	
<1%	25 (36)
1–49%	17 (24)
≥ 50%	17 (24)
Not evaluable**	11 (16)
STAS	28 (40)
Pleural invasion	30 (43)
PL1	16 (23)
PL2	9 (13)
PL3	5 (7)
Vascular invasion	44 (63)
Perineural invasion	8 (11)
Ki‐67 [median (IQR)]	35 (10–60)
WHO grading	
1	1 (2)
2	18 (43)
3	23 (55)
Not evaluable	28 (40)

Abbreviations: MPR, major pathological response; pCR, complete pathological response; PD‐L1, Programmed‐death ligand 1; PL, pleural invasion; STAS, spread through air spaces.

* All data are reported as *N* (%) if not otherwise specified.

** In cases of complete pathological response (pCR) or major pathological response (MPR) with minimal residual tumour cell content, PD‐L1 expression was not assessed.

ALK status was assessed in 45/70 patients (64.3%), with rearrangements identified in 4%. Testing was not applicable in 25.7% (squamous cell carcinomas) and unavailable in 10%.

### Morphometry, Inflammatory Cell Characterization and AI

Morphometrical quantification of the two main stromal components was done on WSI obtained from all patients, stained with Azan Mallory (for fibrosis) or with IHC for CD45 (for inflammation). Fibrosis extension had a median (IQR) of 22% (14%–31%), ranging from 2.4% to 79.9%, and inflammation had a median (IQR) extension of 24% (14%–32%), ranging from 3.6% to 84%. We therefore performed an immunohistochemical characterization of the different inflammatory cell components and quantified the infiltrates with an AI tool on WSI, identifying a median (IQR) of 11% (6%–29%) for CD4+ T lymphocytes, 13% (9%–23%) for CD8+ T lymphocytes, 15% (11%–20%) for CD68+ macrophages, and 6% (4%–10%) for FOXP3+ cells.

### Correlation Between MPR, pCR and Other Pathological Parameters

MPR was associated with a lower frequency of STAS, vascular and pleural invasion (*P* = 0.001, *P* < 0.001 and *P* = 0.012, respectively). As expected, it was also related to the stromal component extension; in particular, MPR was characterized by a higher extension of fibrosis (*P* = 0.004). Moreover, MPR was also associated with lower *ClinPATH combined score* values (all *P* < 0.001).

Similarly, pCR was associated with higher stromal component extension, even if without statistical significance due to the low number of cases. pCR seemed also to be associated with lower *ClinPATH combined scores*.

When the different histotypes were considered separately, adenocarcinomas seemed to have more frequent vascular invasion (*P* = 0.021) and a worse survival, even if without statistical significance.

### Survival Analyses

All clinical‐pathological parameters were evaluated in terms of prognostic significance, both for DFS and OS (Tables 3 and 4).

**Table 3:** Prognostic impact of clinical‐pathological parameters (whole study population)

Parameter	Disease‐free survival HR (95% CI) *P*‐value		Overall survival HR (95% CI) *P*‐value	
*Nominal variables*				
Gender	0.94 (0.52, 1.71)	0.90	0.68 (0.37, 1.25)	0.20
Smoking history	0.58 (0.26, 1.29) 0.86 (0.41, 1.79)	0.20 0.70	0.54 (0.24, 1.22) 0.99 (0.48, 2.07)	0.14 >0.9
ECOG PS at diagnosis	0.76 (0.41, 1.39)	0.40	0.89 (0.48, 1.63)	0.70
Weight loss	0.77 (0.33, 1.81)	0.50	0.94 (0.39, 2.22)	0.90
Clinical stage	1.19 (0.66, 2.14)	0.60	1.20 (0.66, 2.17)	0.60
PD‐L1 expression				
1%–49%	1.13 (0.55–2.32)	0.70	1.13 (0.54, 2.38)	0.70
≥50%	1.39 (0.66–2.92)	0.40	1.84 (0.87, 3.89)	0.11
pCR	0.17 (0.02, 1.20)	**0.075**	0.17 (0.02, 1.22)	**0.077**
MPR				
>10%	3.18 (1.14, 8.87)	**0.027**	2.35 (0.84, 6.57)	0.10
STAS	1.36 (0.75, 2.45)	0.30	1.20 (0.66, 2.19)	0.50
Perineural invasion	2.47 (1.08, 5.64)	**0.03**	2.41 (1.05, 5.52)	**0.037**
Vascular invasion	2.41 (1.24, 4.69)	**0.009**	1.74 (0.90, 3.38)	0.10
Pleural invasion				
1	1.43 (0.70, 2.93)	0.30	1.51 (0.73, 3.13)	0.30
2	1.50 (0.61, 3.69)	0.40	2.05 (0.82, 5.12)	0.12
3	2.78 (1.04, 7.41)	**0.04**	2.28 (0.85, 6.09)	0.10
*Quantitative variables* (*low/high, based on median value* *)				
Age (*68)	1.13 (0.63, 2.02)	0.70	1.13 (0.63, 2.04)	0.70
Neutrophil count at baseline (*5)	1.10 (0.59, 2.04)	0.80	1.24 (0.66, 2.31)	0.50
Lymphocyte count at baseline (*2)	0.59 (0.30, 1.17)	0.13	0.57 (0.29, 1.12)	0.10
Platelet count at baseline (*268)	0.77 (0.41, 1.43)	0.40	0.75 (0.40, 1.41)	0.40
NLR (*3)	0.87 (0.44, 1.69)	0.70	0.91 (0.47, 1.77)	0.80
PLR (*139)	0.74 (0.38, 1.42)	0.40	0.88 (0.45, 1.70)	0.70
Ki‐67 (*35)	1.97 (1.09, 3.57)	**0.025**	2.25 (1.23, 4.11)	**0.009**
Viable tumour cell % (*55)	1.58 (0.88, 2.84)	0.13	1.19 (0.66, 2.15)	0.60
Necrosis % (*5)	0.74 (0.41, 1.34)	0.30	0.81 (0.45, 1.47)	0.50
Stroma % (*30)	0.81 (0.45, 1.45)	0.50	0.77 (0.43, 1.39)	0.40
Fibrosis % (*22)	0.58 (0.32, 1.06)	**0.075**	0.77 (0.42, 1.42)	0.40
Inflammation % (*24)	0.9 (0.5, 1.61)	0.70	0.88 (0.49, 1.59)	0.70
CD4+ T lymphocyte % (*11)	0.94 (0.53, 1.68)	0.80	0.77 (0.42, 1.39)	0.40
CD8+ T lymphocyte % (*13)	0.87 (0.49, 1.56)	0.60	0.81 (0.45, 1.46)	0.50
CD68+ macrophages % (*15)	1.28 (0.71, 2.30)	0.40	1.18 (0.66, 2.13)	0.60
FOXP3+ cells % (*6)	0.78 (0.44, 1.40)	0.40	0.76 (0.42, 1.38)	0.40
*Quantitative variables* (*low/intermediate/high, based on Q1, median and Q3 values* **)				
Ki‐67 (**10, 35, 60)	2.18 (1.08, 4.40) 2.16 (0.94, 4.96)	**0.029** **0.069**	1.62 (0.80, 3.28) 1.84 (0.80, 4.24)	0.20 0.20
Viable tumour cell % (**21, 55, 70)	2.49 (1.09–5.71) 2.56 (0.97–6.73)	**0.031** **0.057**	1.84 (0.80, 4.22) 1.86 (0.7, 4.9)	0.15 0.20
Necrosis % (**1, 5, 19)	1.04 (0.52–2.10) 0.99 (0.43–2.26)	>0.9 >0.9	1.27 (0.62, 2.63) 1.25 (0.54, 2.89)	0.50 0.60
Stroma % (**15, 30, 50)	0.98 (0.51–1.90) 0.53 (0.22–1.27)	>0.9 0.20	0.95 (0.49, 1.84) 0.64 (0.27, 1.54)	0.90 0.30
Fibrosis % (**14, 22, 31)	0.47 (0.25–0.91) 0.35 (0.15–0.83)	**0.024** **0.017**	0.66 (0.34, 1.27) 0.58 (0.24, 1.36)	0.20 0.20
Inflammation % (**14, 24, 32)	0.85 (0.42–1.70) 0.64 (0.29–1.44)	0.60 0.30	0.87 (0.43, 1.77) 0.72 (0.32, 1.61)	0.70 0.40
CD4+ T lymphocyte % (**6, 11, 29)	0.53 (0.26–1.08) 0.65 (0.28–1.49)	0.081 0.30	0.35 (0.17, 0.74) 0.53 (0.23, 1.23)	**0.005** 0.14
CD8+ T lymphocyte % (**9, 13, 23)	0.79 (0.39–1.59) 1.08 (0.50–2.35)	0.50 0.80	0.67 (0.33, 1.36) 0.83 (0.37, 1.85)	0.30 0.60
CD68+ macrophages % (**11, 15, 20)	0.87 (0.43–1.77) 1.68 (0.77–3.68)	0.70 0.20	0.85 (0.42, 1.73) 2.08 (0.95, 4.56)	0.70 0.068
FOXP3+ cells % (**4, 6, 10)	0.75 (0.38–1.47) 0.70 (0.32–1.53)	0.40 0.40	0.70 (0.36, 1.39) 0.59 (0.26, 1.32)	0.30 0.20

Abbreviations: CI, confidence interval; ECOG PS, Eastern Cooperative Oncology Group Performance Status; HR, hazard ratio; NLR, neutrophil‐to‐lymphocyte ratio; MPR, major pathological response; pCR, complete pathological response; PD‐L1, Programmed‐death ligand 1; PLR, platelet‐to‐lymphocyte ratio; STAS, spread through air spaces.

*Note*: Bold *P*‐values are those related to clinico‐pathological variables most impactful for the prognosis.

* Quantitative variables were dichotomized into low and high categories based on the median value.

** Quantitative variables were stratified into low, intermediate and high categories based on the first quartile (Q1), median value and third quartile (Q3).

**Table 4:** Prognostic impact of clinical‐pathological parameters (only adenocarcinomas)

Parameter	Disease‐free survival HR (95% CI) *P*‐value	Overall survival HR (95% CI) *P*‐value		
*Nominal variables*				
Gender	1.32 (0.65, 2.68)	0.40	1.19 (0.57, 2.47)	0.60
Smoking history	0.54 (0.20, 1.41) 0.88 (0.39, 2.00)	0.20 0.80	0.59 (0.22, 1.56) 0.90 (0.39, 2.10)	0.30 0.80
ECOG PS at diagnosis	0.85 (0.40, 1.80)	0.70	0.89 (0.42, 1.91)	0.80
Weight loss	1.34 (0.55, 3.29)	0.50	1.66 (0.66, 4.17)	0.30
Clinical stage	3.88 (1.81, 8.30)	**<0.001**	3.03 (1.45, 6.34)	**0.003**
PD‐L1 expression				
1%–49%	1.36 (0.55, 3.37)	0.50	2.01 (0.80, 5.09)	0.14
≥50%	1.43 (0.56, 3.63)	0.50	1.71 (0.68, 4.29)	0.30
pCR	0.50 (0.07, 3.70)	0.50	0.44 (0.06, 3.22)	0.40
MPR				
>10%	2.57 (0.78, 8.47)	0.12	1.63 (0.49, 5.41)	0.40
STAS	0.95 (0.46, 1.93)	0.90	0.62 (0.29, 1.29)	0.20
Perineural invasion	3.14 (0.88, 11.1)	**0.077**	5.43 (1.42, 20.8)	**0.014**
Vascular invasion	3.36 (1.28, 8.83)	**0.014**	2.22 (0.85, 5.81)	0.11
Pleural invasion	1.61 (0.73, 3.57) 1.62 (0.53, 4.96) 4.48 (0.96, 20.9)	0.20 0.40 **0.056**	1.75 (0.77, 3.98) 2.10 (0.67, 6.55) 4.67 (0.98, 22.2)	0.20 0.20 **0.053**
WHO grading	2.45 (1.15, 5.20)	**0.02**	1.89 (0.89, 4.01)	0.10
*Quantitative variables* (*low/high, based on median value* *)				
Age (*68)	1.18 (0.58, 2.40)	0.70	0.97 (0.47, 2.00)	>0.9
Neutrophil count at baseline (*5)	1.18 (0.56, 2.49)	0.70	1.34 (0.62, 2.86)	0.50
Lymphocyte count at baseline (*2)	0.25 (0.10, 0.66)	**0.005**	0.30 (0.12, 0.74)	**0.009**
Platelet count at baseline (*268)	0.94 (0.43, 2.05)	0.90	1.21 (0.55, 2.65)	0.60
NLR (*3)	1.98 (0.88, 4.44)	0.10	2.17 (0.95, 4.93)	**0.065**
PLR (*139)	1.21 (0.55, 2.66)	0.60	1.97 (0.82, 4.70)	0.13
Ki‐67 (*35)	2.71 (1.32, 5.58)	**0.007**	2.51 (1.20, 5.23)	**0.014**
Viable tumour cell % (*55)	1.09 (0.54, 2.23)	0.80	0.66 (0.31, 1.39)	0.30
Necrosis % (*5)	1.07 (0.52, 2.18)	0.90	0.86 (0.40, 1.84)	0.70
Stroma % (*30)	0.91 (0.45, 1.84)	0.80	1.18 (0.56, 2.47)	0.70
Fibrosis % (*22)	0.47 (0.22, 1.00)	**0.051**	0.81 (0.37, 1.76)	0.60
Inflammation % (*24)	0.83 (0.41, 1.69)	0.60	0.97 (0.47, 2.01)	>0.9
CD4+ T lymphocyte % (*11)	1.01 (0.50, 2.05)	>0.9	0.81 (0.39, 1.69)	0.60
CD8+ T lymphocyte % (*13)	0.83 (0.41, 1.70)	0.60	0.86 (0.42, 1.78)	0.70
CD68+ macrophages % (*15)	0.98 (0.47, 2.01)	>0.9	0.81 (0.39, 1.68)	0.60
FOXP3+ cells % (*6)	0.39 (0.18, 0.83)	**0.015**	0.46 (0.22, 0.96)	**0.038**
*Quantitative variables* (*low/intermediate/high, based on Q1, median and Q3 values* **)				
Ki‐67 (**10, 35, 60)	2.21 (0.99, 4.94) 5.41 (1.89, 15.5)	**0.054** **0.002**	1.31 (0.57, 2.99) 3.85 (1.37, 10.8)	0.50 **0.011**
Viable tumour cell % (**21, 55, 70)	2.46 (0.91, 6.64) 1.89 (0.60, 6.01)	**0.075** 0.30	1.83 (0.68, 4.91) 0.79 (0.22, 2.75)	0.20 0.70
Necrosis % (**1, 5, 19)	0.75 (0.33, 1.72) 1.62 (0.57, 4.59)	0.50 0.40	0.69 (0.30, 1.61) 1.87 (0.66, 5.31)	0.40 0.20
Stroma % (**15, 30, 50)	0.95 (0.41, 2.20) 0.40 (0.13, 1.23)	>0.9 0.11	1.77 (0.69, 4.55) 0.92 (0.28, 3.04)	0.20 0.90
Fibrosis % (**14, 22, 31)	0.34 (0.16, 0.75) 0.28 (0.10, 0.80)	**0.008** **0.017**	0.59 (0.26, 1.33) 0.73 (0.24, 2.16)	0.20 0.60
Inflammation % (**14, 24, 32)	0.74 (0.32, 1.73) 0.81 (0.33, 2.00)	0.50 0.60	1.03 (0.43, 2.42) 1.20 (0.48, 3.04)	>0.9 0.70
CD4+ T lymphocyte % (**6, 11, 29)	0.61 (0.25, 1.51) 0.96 (0.32, 2.88)	0.30 >0.9	0.47 (0.19, 1.15) 0.90 (0.30, 2.71)	0.10 0.90
CD8+ T lymphocyte % (**9, 13, 23)	0.46 (0.19, 1.11) 1.40 (0.51, 3.80)	0.083 0.50	0.38 (0.16, 0.92) 1.85 (0.68, 5.07)	**0.032** 0.20
CD68+ macrophages % (**11, 15, 20)	0.62 (0.27, 1.44) 1.02 (0.40, 2.60)	0.30 >0.9	0.51 (0.21, 1.20) 1.16 (0.46, 2.90)	0.12 0.80
FOXP3+ cells % (**4, 6, 10)	0.55 (0.25, 1.22) 0.34 (0.12, 0.96)	0.14 **0.042**	0.49 (0.21, 1.11) 0.26 (0.09, 0.77)	0.087 **0.015**

Abbreviations: CI, confidence interval; ECOG PS, Eastern Cooperative Oncology Group Performance Status; HR, hazard ratio; MPR, major pathological response; NLR, neutrophil‐to‐lymphocyte ratio; pCR, complete pathological response; PD‐L1, Programmed‐death ligand 1; PLR, platelet‐to‐lymphocyte ratio; STAS, spread through air spaces.

*Note*: Bold *P*‐values are those related to clinico‐pathological variables most impactful for the prognosis.

* Quantitative variables were dichotomized into low and high categories based on the median value.

** Quantitative variables were stratified into low, intermediate and high categories based on the first quartile (Q1), median value and third quartile (Q3).

Concerning routine pathologic assessment, MPR and pCR were associated with a longer DFS (HR = 3.18, 95% CI: 1.14–8.87, *P* = 0.027 and HR = 0.17, 95% CI: 0.02, 1.20), *P* = 0.075, respectively. When remaining viable tumour cells were considered as a continuous variable, intermediate/high values were confirmed to be associated with a worse DFS (*P* = 0.031/*P* = 0.057). Necrosis and stromal extension percentages were not associated with prognosis.

**Figure 1:**
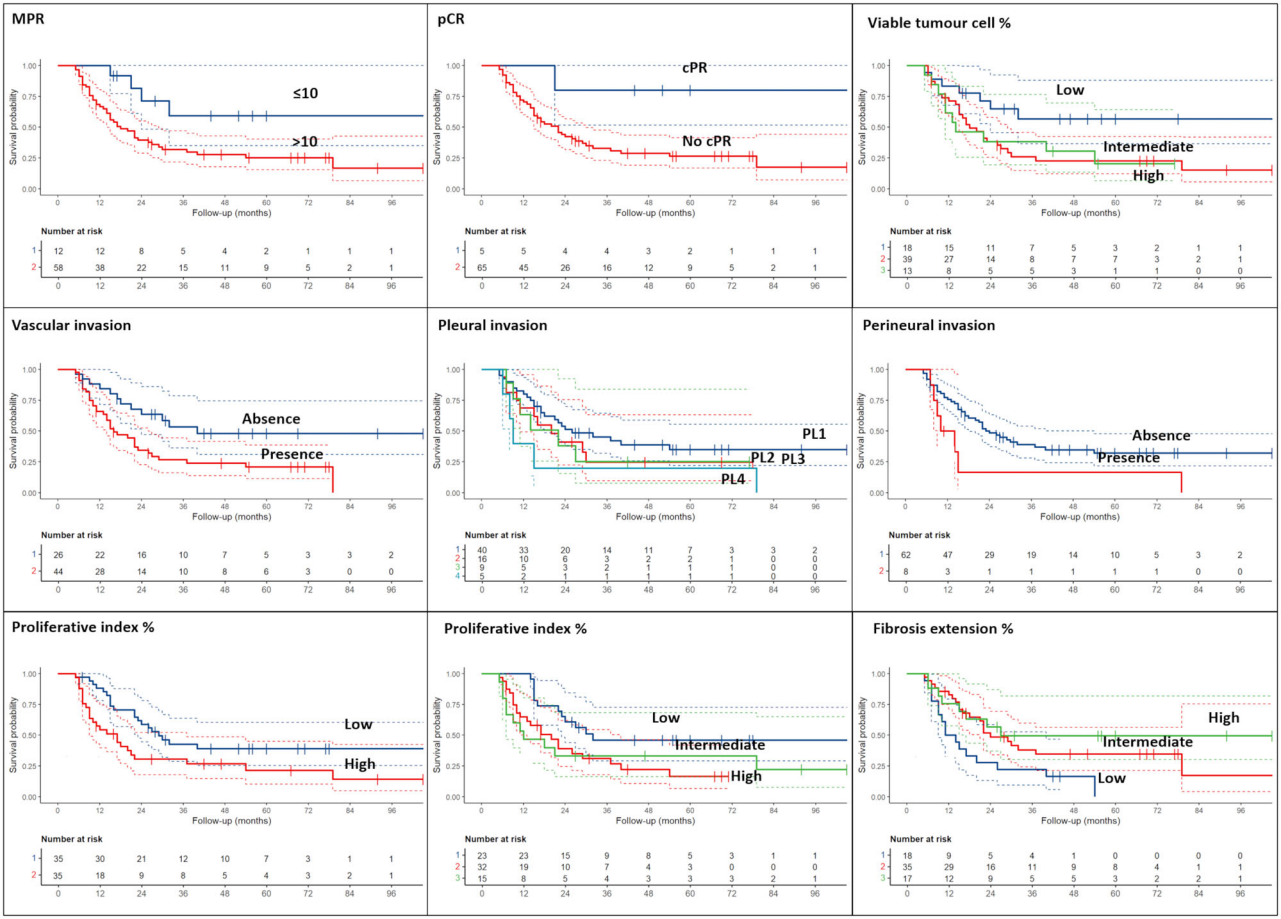
Disease‐free survival data and curves according to the most significant clinical‐pathological parameters.

Morphometrical quantification of the stromal components highlighted that intermediate/high fibrosis extension was associated with a longer DFS (*P* = 0.024/*P* = 0.017), while inflammation percentage continued to be not prognostic. In addition, shorter DFS was correlated with the presence of perineural invasion (*P* = 0.03), vascular invasion (*P* = 0.009), pleural invasion (*P* = 0.04 for PL3) and higher Ki‐67 (*P* = 0.025) (Table 3, Figure 1).

When focusing on OS, pCR was confirmed to be associated with a better prognosis (*P* = 0.077), while MPR did not show a statistically significant relation (HR = 2.35, 95% CI: 0.84–6.57, *P* = 0.10). The presence of perineural invasion and high ki‐67 was associated with a worse survival rate (*P* = 0.037 and *P* = 0.009). Among inflammatory cells, only CD4+ T cell count trends with survival: higher CD4+ T cell levels were associated with improved OS (*P* = 0.005 for intermediate and *P* = 0.14 for high values).

**Figure 2:**
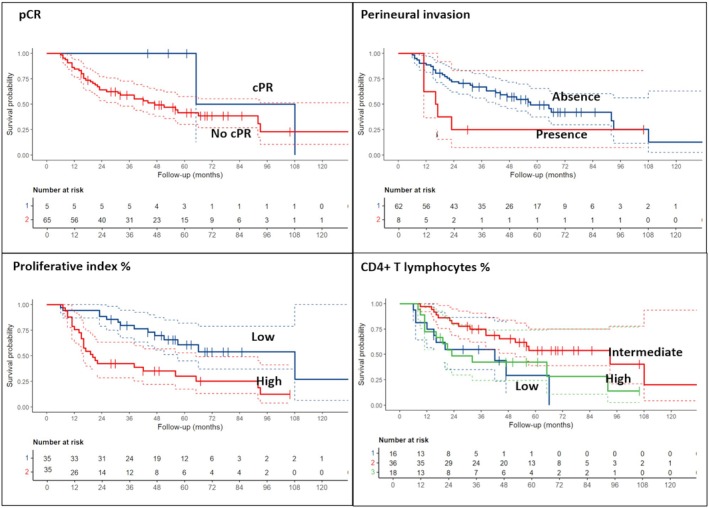
Overall survival data and curves according to the most significant clinical‐pathological parameters.

No other parameters, including PD‐L1, were significantly associated with patient prognosis (Table 3, Figure 2).

**Figure 3:**
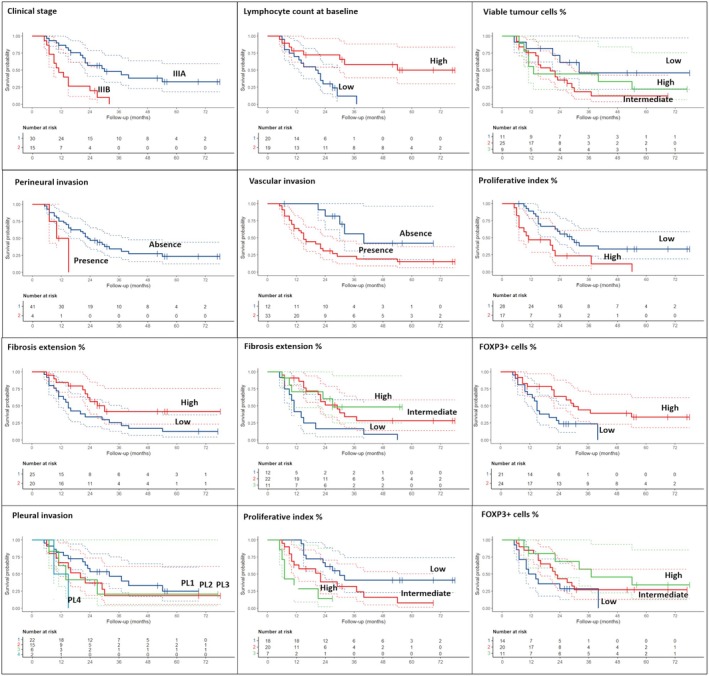
Disease‐free survival data and curves according to the most significant clinical‐pathological parameters in patients with adenocarcinoma.

**Figure 4:**
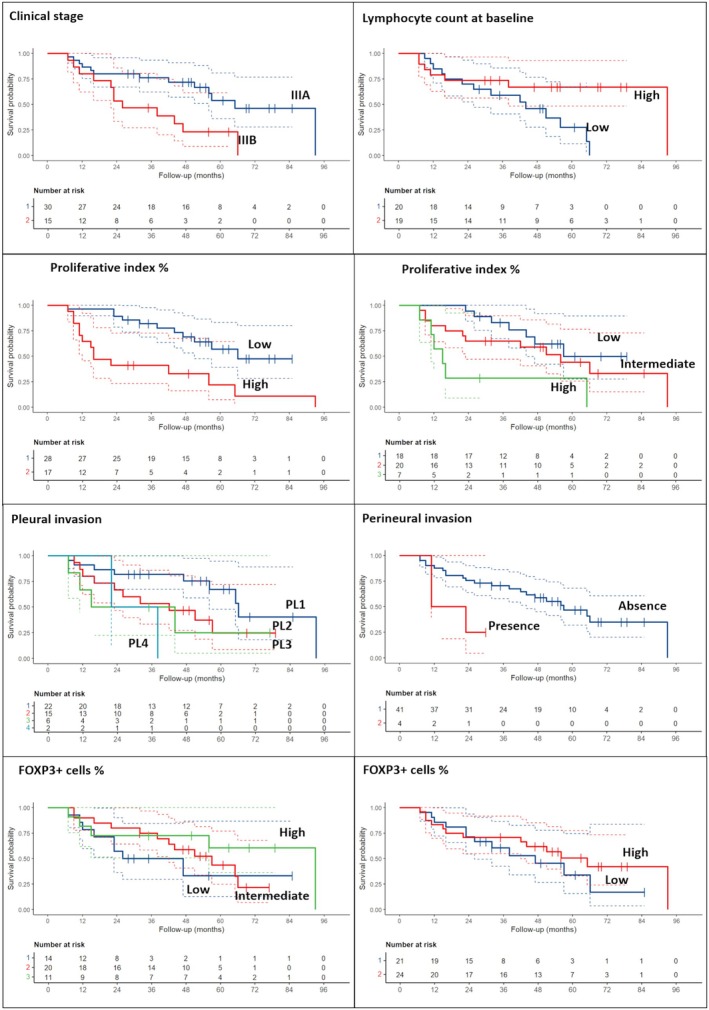
Overall survival data and curves according to the most significant clinical‐pathological parameters in patients with adenocarcinoma.

When considering only adenocarcinomas, clinical stage IIIB and the low number of blood lymphocytes at the baseline were associated with a shorter DFS (*P* < 0.001 and *P* = 0.005, respectively). In addition, different pathological features were related to a worse patient prognosis, such as the presence of vascular invasion (*P* = 0.014), perineural invasion (*P* = 0.077), pleural invasion (*P* = 0.056 for PL3), higher WHO grading (*P* = 0.02) and high Ki‐67 values (*P* = 0.007). Higher fibrosis extension and FOXP3+ cell percentage were related to a longer DFS (*P* = 0.051 and *P* = 0.015, respectively) (Table 4, Figure 3). Focusing on OS, almost all those parameters confirmed the prognostic role (Table 4, Figure 4). No other parameters, including PD‐L1, were significantly associated with patient prognosis.

**Figure 5:**
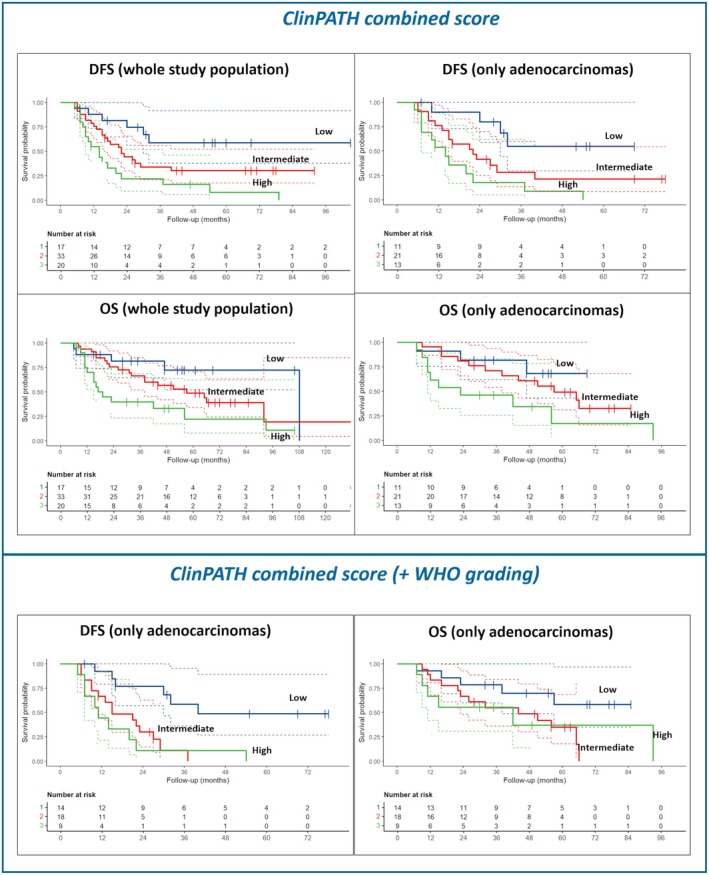
(**A**, **C**) Disease‐free survival data and curves according to the *ClinPATH combined score B*, in the whole study population (**A**) and when considering only adenocarcinomas (**C**). (**B**, **D**) Overall survival data and curves according to the *ClinPATH combined score* B, in the whole study population (**B**) and when considering only adenocarcinomas (**D**).

**Figure 6:**
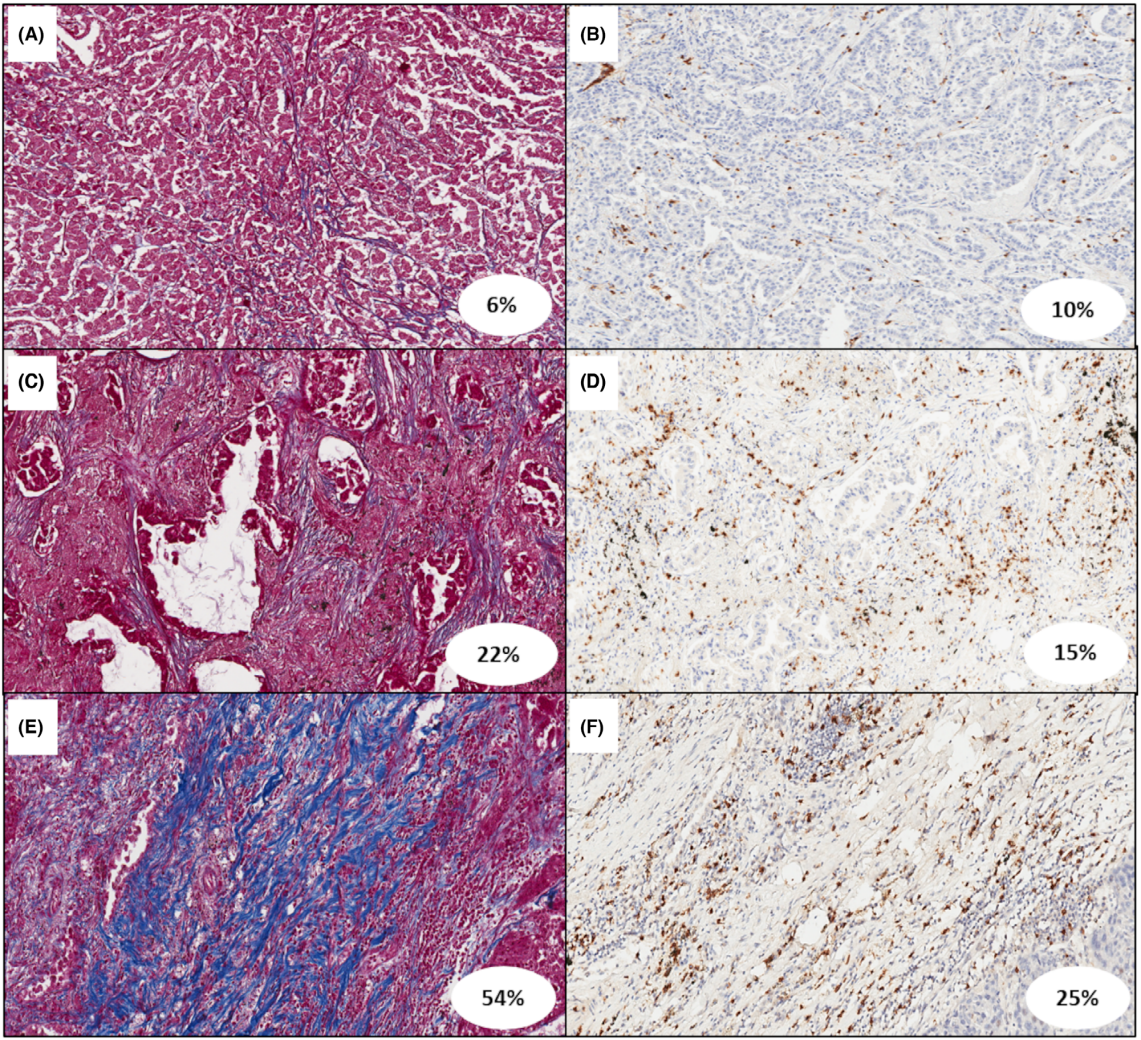
Explanatory images of Azan‐Mallory stain (**A**, **C**, **E**) and immunohistochemistry for CD4+ T lymphocytes (**B**, **D**, **F**).

We then explored the prognostic impact of different scores with the most impactful clinical/pathological parameters combined (ClinPATH), considering the whole study population or just adenocarcinomas alone (Table 5). The best combination for predicting patient prognosis was found in the *ClinPATH combined score ‘B’*. Indeed, the *ClinPATH combined score* B—which incorporates MPR, fibrosis extension, CD4^+^ T cell percentage and other selected features—demonstrated significantly stronger associations with both DFS (HR = 6.47, 95% CI: 2.67–15.6, *P* < 0.001) and OS (HR = 5.09, 95% CI: 2.12–12.2, *P* < 0.001) (Table 6, Figure 5). Interestingly, when analyses were only limited to adenocarcinomas, the performance of *ClinPATH combined score ‘B’* significantly increased (Table 6, Figure 5). An explanatory case is shown in Figure 6.

**Table 5:** ClinPATH combined scores

ClinPATH scores	Variable	Attributed points	Score
A	Lymphocyte count at baseline		
	Low (≤2)	1	0–1
	High (>2)	0	
	Perineural invasion		
	Presence	1	0–1
	Absence	0	
	Vascular invasion		
	Presence	1	0–1
	Absence	0	
	Ki‐67		
	Low (≤35)	0	0–1
	High (>35)	1	
	Fibrosis %		
	Low (≤14)	2	0–2
	Intermediate (15–31)	1	
	High (>31)	0	
	MPR		
	≤10%	0	0–1
	>10%	1	
	**Added variables**		
B	CD4+ T lymphocytes %		
	High (>11)	0	0–1
	Low (≤11)	1	
C	CD4+ T lymphocytes %		
	High (>11)	0	0–1
	Low (≤11)	1	
	FOXP3+ cell %		
	High (>6)	0	0–1
	Low (≤6)	1	
	**Added variables (only for adenocarcinomas)**		
A1, B1, C1	WHO grading (only for adenocarcinomas)		
	1	0	0–2
	2	1	
	3	2	

ClinPATH composite scores were categorized according to their empirical distribution, using quartiles and the median as reference thresholds. Score A (0–7) was classified as low 0–2, intermediate 3–4, high 5–7; Score B (0–8) as low 0–3, intermediate 4–5, high 6–8; and Score C (0–9) as low 0–3, intermediate 4–5, high 6–9.

Abbreviations: MPR, major pathological response; WHO, World Health Organization.

**Table 6:** Prognostic impact of the *ClinPATH combined scores*

	Disease‐free survival HR (95% CI) *P*‐value		Overall survival HR (95% CI) *P*‐value	
*ClinPATH combined score A* *				
Whole population (70 patients)	2.44 (0.99, 6.04)	**0.054**	1.67 (0.67, 4.16)	0.30
	4.76 (1.87, 12.1)	**0.001**	3.97 (1.57, 10.1)	**0.004**
Only adenocarcinomas (45 patients)	2.96 (0.98, 8.99)	**0.055**	1.37 (0.45, 4.18)	0.60
	5.83 (1.85, 18.4)	**0.003**	3.36 (1.06, 10.7)	**0.04**
*ClinPATH combined score B* **				
Whole population (70 patients)	2.78 (1.29, 5.98)	**0.009**	2.14 (0.99, 4.61)	**0.053**
	6.47 (2.67, 15.6)	**<0.001**	5.09 (2.12, 12.2)	**<0.001**
Only adenocarcinomas (45 patients)	2.92 (1.18, 7.20)	**0.02**	1.58 (0.63, 3.92)	0.30
	11.1 (3.71, 33.0)	**<0.001**	8.82 (3.00, 26.0)	**<0.001**
*ClinPATH combined score C* ***				
Whole population (70 patients)	2.31, 0.99–5.40	**0.053**	1.77 (0.76, 4.14)	0.20
	5.37, 2.18–13.2	**<0.001**	4.15 (1.69, 10.2)	**0.002**
Only adenocarcinomas (45 patients)	2.55 (0.91, 7.15)	0.074	1.45 (0.51, 4.13)	0.50
	14.7 (4.46, 48.7)	**<0.001**	5.62 (1.95, 16.2)	**0.001**
*ClinPATH combined score A1* ****				
Only adenocarcinomas (45 patients)	4.52 (1.64, 12.4)	**0.003**	2.82 (1.08, 7.36)	**0.035**
	5.92 (2.06, 17.0)	**< 0.001**	3.99 (1.36, 11.7)	**0.012**
*ClinPATH combined score B1* ****				
Only adenocarcinomas (45 patients)	1.49 (0.52, 4.32)	0.50	1.26 (0.43, 3.67)	0.70
	5.74 (2.01, 16.4)	**<0.001**	2.54 (0.90, 7.14)	0.077
*ClinPATH combined score C1* ****				
Only adenocarcinomas (45 patients)	2.99 (0.66, 13.6)	0.20	2.00 (0.44, 9.06)	0.40
	9.22 (2.07, 40.9)	**0.004**	3.70 (0.85, 16.2)	0.082

Abbreviations: CI, confidence interval; HR, hazard ratio; WHO, World Health Organization.

*Note*: Bold *P*‐values are those related to clinico‐pathological variables most impactful for the prognosis.

* *ClinPATH combined score* A includes MPR, baseline blood lymphocytes, perineural invasion, vascular invasion, proliferative index and fibrosis extension percentage.

** *ClinPATH combined score* B includes MPR, baseline blood lymphocytes, perineural invasion, vascular invasion, proliferative index, fibrosis extension percentage and AI‐quantified CD4+ cell %.

*** *ClinPATH combined score* C includes MPR, baseline blood lymphocytes, perineural invasion, vascular invasion, proliferative index, fibrosis extension percentage, AI‐quantified CD4+ cell % and FOXP3+ cell %.

**** *ClinPATH combined scores* A1, B1, C1 are obtained by adding WHO grading to *ClinPATH combined scores* A, B, C, respectively.

## Discussion

Currently, the pathological assessment of resected lung cancer following neoadjuvant therapy represents a growing area of interest, particularly with a rise in therapeutic strategies such as targeted and immune therapies over time. To the best of our knowledge, this is the first study that has investigated the response to NACT considering a wide comprehensive histopathological evaluation of the lung tumour specimens and a morphometrical/AI‐mediated quantification of the tumour bed stromal components.

Until now, the only prognostically significant parameter after NACT is represented by the residual viable tumour cell percentage, usually reported as MPR and/or pCR^5^, ^11^, ^19^, ^20^, ^21^, ^22^, ^23^, ^24^, ^25^, ^26^, ^27^ and this was also confirmed in our case series. In our study, the other tumour bed components (stroma and necrosis), evaluated following IASLC recommendations, did not seem to have an impact on the patient prognosis. Nevertheless, our data show that MPR alone had limited prognostic value for OS in this cohort, with only borderline statistical significance. On the other hand, the integration of multiple biologically meaningful variables into the ClinPATH score—despite not being part of standard practice (e.g. fibrosis extent, CD4^+^ infiltration)—improved the prediction of both DFS and OS. These findings support the rationale for incorporating advanced pathological and AI‐based features into composite prognostic tools, especially if supported by future external validation.

However, when the stromal components were systematically analysed by computer‐assisted morphometry, a higher fibrosis extension was detected as an important prognostic factor, correlating with a better survival rate.

This result is in line with another study that analytically scored the fibrotic response to NACT in the case of pancreatic cancer. The authors hypothesized the increased posttreatment fibrosis as a surrogate measure of treatment response, providing information about patient prognosis.^28^ This result could be partially expected, as fibrosis is inversely correlated with the presence of viable tumour cells. However, Cox univariate regression analysis showed that the extent of fibrosis appeared to have a greater prognostic impact than the residual tumour cells. Moreover, the inclusion of both MPR and fibrosis in the *ClinPATH combined score* resulted in improved performance in survival prediction. Moreover, we explored the prognostic role of additional morphological parameters, and we identified perineural, vascular and pleural invasion as predictors of a worse DFS and OS, particularly when the analysis was restricted to adenocarcinoma. An interesting study by Qu *et al*. focused on the evaluation of some histological findings after NACT, and lymphovascular invasion was already found associated with lung cancer‐specific death, particularly when SCC samples were considered.^11^ Our findings are also in line with evidence in other organs.^29^, ^30^

In addition, proliferative activity was inversely related to DFS and OS; indeed, patients with higher Ki‐67+ cell percentages had a doubling in the risk of both recurrence and death in the whole study population, overcoming a fourfold increased risk of recurrence and a fivefold risk of death when only adenocarcinoma cases were considered. In the literature, the prognostic role of the Ki‐67 proliferative index is contradictory, particularly in the NACT setting.^31^

Focusing on the AI quantification of inflammatory cells, higher CD4+ T lymphocyte and FOXP3+ cell percentages trend with survival, while the other cells did not seem to have a prognostic role.

This result is in line with other studies that highlighted that high tumour stroma infiltrating Foxp3+ and CD4+ T cells were independently associated with improved NSCLC patient survival rates.^32^, ^33^

Among clinical‐laboratory data, only the clinical stage and the lymphocyte cell count at baseline resulted predictive of the outcome, particularly when the analyses were restricted to adenocarcinoma. To date, only a few studies have investigated the prognostic role of haematological values and indices in the neoadjuvant setting. Sun *et al*. highlighted a correlation between neutrophil‐to‐lymphocyte (NLR) ratio and a shorter DFS in patients with NSCLC undergoing NACT or combined with PD‐1 checkpoint inhibitors.^34^ Other studies showed similar results focusing only on neoadjuvant immunotherapy, supporting the use of NLR as a predictor of efficacy.^35^, ^36^, ^37^

The combination of most impactful clinical‐pathological variables into a *ClinPATH combined score* leads to better prognostic stratification of the study population, both in terms of DFS and OS.

To date, different prognostic models that include both clinical and pathological parameters have been proposed for locally advanced NSCLC, but only in a few cases in the NACT setting.^38^, ^39^, ^40^ To the best of our knowledge, only the Prognostic Score for Residual Cancer (PRSC) was reported to predict survival in NSCLC after NACT, and it included T‐category, lymph node status and MPR,^26^ even if the validation in an independent cohort of patients showed that PRSC was not superior compared with MPR alone.^5^

A limitation of the current investigation is its retrospective nature. Another limitation is the use of computer‐assisted morphometry and AI in the most representative slide of the tumour bed. As this approach is expensive and time‐consuming, we aimed to test if the evaluation of only one slide could be efficient and potentially included in the diagnostic routine, and we verified that the most representative section could really be considered the mirror of the whole tumour bed.

Concerning the AI algorithm, a formal model training and independent validation were not performed in this study, but the application was designed in close collaboration with Visiopharm's scientific support team to ensure technical accuracy and suitability for our histological data. We have planned to validate the same algorithm on a large independent cohort of NSCLC tumours. The ClinPATH score surely requires a more labour‐intensive assessment; however, it may also pave the way for targeted therapies aimed at those components of the TME that were found to have the greatest prognostic impact (fibrosis and CD4+ T lymphocyte infiltration). Nevertheless, if validated in larger, multicentre independent cohorts, it could become feasible in routine practice with the support of dedicated AI‐based tools specifically designed to quantify fibrosis extension and CD4+ T lymphocyte infiltration.

We are also aware that the immunophenotyping approach was not exhaustive and the spatial distribution of the immune infiltrate (i.e. intra‐ vs. peritumoral localization) was not assessed, and these aspects merit further studies, particularly if immunotherapy is considered as a neoadjuvant approach. Finally, we acknowledge that excluding lymph nodes from the tumour bed assessment may represent a limitation, particularly in light of the emerging interest in evaluating nodal response after neoadjuvant therapy, and this aspect will be the focus of future dedicated studies.

In order to validate and improve our results, even overcoming the limitations of the retrospective nature of this study, we are planning a prospective phase including locally advanced NSCLC patients judged to be potentially resectable after NACT combined with immunotherapy. This study may be useful in identifying predictive and prognostic factors in this particular setting.

## Conclusion

In summary, our study confirmed MPR and pCR as good prognostic factors, but the combination of the most impactful clinico‐pathological parameters correlated better with DFS and OS than any individual parameter. These results could be highly valuable for clinicians in guiding therapeutic decisions, helping oncologists tailor adjuvant treatments, optimize follow‐up strategies and ultimately improve patient outcomes.

## Author contributions

F.L., G.P. and F.C. designed the research study. F.L., F.P., S.T., A.K., Y.K. and S.Z. performed the research. M.S., F.R., A.F. and G.P. followed the patients and collected clinical data. L.V. analysed the data, and F.L. and F.C. wrote the paper.

## Funding information

This work was supported by the Italian Ministry of University and Research (2022SLM9AN) and by the University of Padova (IRON 2021‐89).

## Conflict of interest

None declared.

## Data Availability

All data are available upon previous request to the authors.
